# Outcome after neoadjuvant chemotherapy in Asian breast cancer patients

**DOI:** 10.1002/cam4.985

**Published:** 2016-12-20

**Authors:** Li Yan Lim, Hui Miao, Joline S. J. Lim, Soo Chin Lee, Nirmala Bhoo‐Pathy, Cheng Har Yip, Nur Aishah B. M. Taib, Patrick Chan, Ern Yu Tan, Swee Ho Lim, Geok Hoon Lim, Evan Woo, Yia Swam Tan, Jung Ah Lee, Mabel Wong, Puay Hoon Tan, Kong Wee Ong, Fuh Yong Wong, Yoon Sim Yap, Mikael Hartman

**Affiliations:** ^1^ Department of SurgeryNational University Health System1E Kent Ridge RoadSingapore119228Singapore; ^2^ Saw Swee Hock School of Public HealthNational University of SingaporeTahir Foundation Building, 12 Science Drive 2Singapore117549Singapore; ^3^ Department of Hematology OncologyNational University Health System1E Kent Ridge RoadSingapore119228Singapore; ^4^ Julius Centre University MalayaFaculty of MedicineUniversity of MalayaKuala Lumpur50603Malaysia; ^5^ Department of SurgeryFaculty of MedicineUniversity of MalayaKuala Lumpur50603Malaysia; ^6^ Department of SurgeryTan Tock Seng Hospital11 Jalan Tan Tock SengSingapore308433Singapore; ^7^ Breast DepartmentKK Women's and Children's Hospital100 Bukit Timah RoadSingapore229899Singapore; ^8^ Department of Medical OncologyNational Cancer Centre Singapore11 Hospital DriveSingapore169610Singapore; ^9^ Department of PathologySingapore General Hospital20 College RoadSingapore169856Singapore; ^10^ Division of Surgical OncologyNational Cancer Centre Singapore11 Hospital DriveSingapore169610Singapore; ^11^ Division of Radiation OncologyNational Cancer Centre Singapore11 Hospital DriveSingapore169610Singapore; ^12^ Department of SurgeryNational University of Singapore and National University Health System1E Kent Ridge RoadSingapore119228Singapore

**Keywords:** Breast cancer, clinicopathologic predictors, neoadjuvant chemotherapy, pathologic complete response

## Abstract

We aim to identify clinicopathologic predictors for response to neoadjuvant chemotherapy and to evaluate the prognostic value of pathologic complete response (pCR) on survival in Asia. This study included 915 breast cancer patients who underwent neoadjuvant chemotherapy at five public hospitals in Singapore and Malaysia. pCR following neoadjuvant chemotherapy was defined as 1) no residual invasive tumor cells in the breast (ypT0/is) and 2) no residual invasive tumor cells in the breast and axillary lymph nodes (ypT0/is ypN0). Association between pCR and clinicopathologic characteristics and treatment were evaluated using chi‐square test and multivariable logistic regression. Kaplan–Meier analysis and log‐rank test, stratified by other prognostic factors, were conducted to compare overall survival between patients who achieved pCR and patients who did not. Overall, 4.4% of nonmetastatic patients received neoadjuvant chemotherapy. The median age of preoperatively treated patients was 50 years. pCR rates were 18.1% (pCR ypT0/is) and 14.4% (pCR ypT0/is ypN0), respectively. pCR rate was the highest among women who had higher grade, smaller size, estrogen receptor negative, human epidermal growth factor receptor 2‐positive disease or receiving taxane‐based neoadjuvant chemotherapy. Patients who achieved pCR had better overall survival than those who did not. In subgroup analysis, the survival advantage was only significant among women with estrogen receptor‐negative tumors. Patients with poor prognostic profile are more likely to achieve pCR and particularly when receiving taxane‐containing chemotherapy. pCR is a significant prognostic factor for overall survival especially in estrogen receptor‐negative breast cancers.

## Introduction

Neoadjuvant chemotherapy is offered to breast cancer patients with inoperable tumors or tumors that are too large for breast conservation, in order to allow for possible resection or breast conservation, respectively 1. It provides comparable survival benefits to adjuvant chemotherapy for breast cancer 2, 3, 4, 5. Pathologic complete response (pCR), which is associated with excellent long‐term prognosis, was reported to be up to 45.8% when definition of pCR was taken as absence of invasive tumor in the breast but allow for in situ tumor 6, 7. pCR ranges from 12% to 19.4% across various study populations when defined as no residual invasive or in situ tumor in the breast and axillary lymph nodes 8, 9.

In most Asian countries, breast cancer rates have been on the rise over the past two decades 10, 11, 12, 13 and these Asian women present to a large extent with more advanced disease 14. Given that Asian women present with larger tumors, neoadjuvant chemotherapy plays an even more important role. Most large multi‐center studies are done in the United States, Europe, and Australia 15, 16, with few done specifically in Asia. Varying use of fourth‐generation chemotherapy as well as trastuzumab for human epidermal growth factor receptor 2 (HER2)‐positive disease were reported in published studies 6, 16, 17, 18. Given the above difference in epidemiology of breast cancer patients in Asia as compared to non‐Asian patients, we aim to identify clinicopathologic and therapeutic predictors for response to neoadjuvant chemotherapy and evaluate the prognostic value of pCR on overall survival in a multi‐ethnic Asian setting.

## Materials and Methods

A total of 915 nonmetastatic breast cancer patients, who underwent neoadjuvant chemotherapy and subsequently had breast surgery, were identified from four public tertiary hospitals in Singapore and one tertiary hospital in Malaysia, namely National University Hospital (NUH), National Cancer Centre Singapore (NCCS), Tan Tock Seng Hospital (TTSH), KK Women's and Children's Hospital (KKH), and University Malaya Medical Centre (UMMC). The hospitals started their hospital‐based breast cancer registries in different years, with the years of diagnosis of the patients between 1993 and 2013. This study was approved by National Healthcare Group Domain Specific Review Board, SingHealth Centralised Institutional Review Board, and UMMC Medical Ethics Committee.

Clinicopathologic information such as tumor grade, estrogen receptor (ER), progesterone receptor (PR) and HER2 status, clinical tumor size, clinical lymph node status and histological type were collected at all five hospitals using a standardized form. Basic patient demographics such as age at diagnosis and ethnicity were included. Tumor grade was evaluated according to the Elston–Ellis modification of Scarff–Bloom–Richardson grading system. If pretreatment biopsy tumor grade was not available, posttreatment grade was recorded, although it is noted that the latter may not accurately reflect original grade due to neoadjuvant chemotherapy effect. ER and PR status were determined via immunohistochemical staining either during core biopsies or using specimen from operation. Positive hormonal receptor status was deemed when 1% or more cells stained positive at NUH or 10% or more positively stained tumor cells at all other hospitals. HER2 status was based on fluorescence in situ hybridization (FISH) or immunohistochemistry (IHC) if FISH was not performed. HER2 positive was defined as FISH positive or IHC score of 3+, negative was defined as FISH negative or IHC scored of 0 or 1+, while equivocal was defined as IHC score of 2+ without confirmatory FISH test. For HER2 status, the data were not available before mid‐2000 for NUH and the completeness of this variable for UMMC is lower across the study period. All breast cancers were staged according to the 7th edition of TNM classification by American Joint Committee on Cancer (AJCC) 19. Treatment data consisted consist of type of type of neoadjuvant chemotherapy regimens (taxane containing vs. nontaxane containing) as well as type of surgery (mastectomy or breast‐conserving surgery). Use of preoperative anti‐HER2 therapy was only systematically recorded in registries at KKH and NCCS. Outcomes postneoadjuvant chemotherapy included size of invasive residual tumors resected, number of lymph nodes resected, and number of lymph nodes involved with tumor. All the databases from the five hospitals were subsequently merged.

Two definitions of pCR to neoadjuvant chemotherapy were used in this paper. The first definition of pCR (pCR (ypT0/is) in Table 1) requires no invasive residual tumors in the breast but allows for in situ disease, regardless of pathologic nodal status 20, 21. In the second definition, pCR (pCR (ypT0/is ypN0) in Table 1) is defined as no invasive residual disease in both breast and axillary lymph nodes but allows for in situ disease, as patients who are found to have invasive residual disease in the nodes with complete response in the breast have worse prognosis than those who had pCR in both breast and nodes 22, 23.

**Table 1:** Demographics, clinicopathologic information, and treatments of breast cancer patients who underwent neoadjuvant chemotherapy at five public hospitals in Singapore and Malaysia (*N* = 915)

No. of neoadjuvant cases	KKH103	NUH181	NCCS302	TTSH137	UMMC192	Total915
Year of diagnosis	2005–2013	2002–2010	2000–2012	2005–2013	1993–2010	1993–2013
Median follow‐up time (months)	36	57.5	33	34	32	38
pCR (ypT0/is)						
Yes	1918.4%	147.7%	5618.5%	2216.1%	2513.0%	13614.9%
No	7673.8%	15585.6%	23176.5%	10073.0%	5026.0%	61266.9%
Unknown	87.8%	126.6%	155.0%	1510.9%	11760.9%	16718.3%
pCR(ypT0/is ypN0)						
Yes	1615.5%	126.6%	5217.2%	1913.9%	2211.5%	12113.2%
No	8279.6%	16189.0%	24179.8%	10677.4%	12464.6%	71478.0%
Unknown	54.9%	84.4%	93.0%	128.8%	4624.0%	808.7%
Age						
<=34	1110.7%	105.5%	124.0%	85.8%	2513.0%	667.2%
35–44	2221.4%	3821.0%	5417.9%	2518.2%	5528.6%	19421.2%
45–54	3937.9%	7541.4%	12441.1%	4835.0%	7338.0%	35939.2%
55–64	2019.4%	4625.4%	8528.1%	3827.7%	3116.1%	22024.0%
65–74	87.8%	116.1%	278.9%	1611.7%	84.2%	707.7%
>=75	32.9%	00.0%	00.0%	21.5%	00.0%	50.5%
Unknown	00.0%	10.6%	00.0%	00.0%	00.0%	10.1%
Ethnicity						
Chinese	7068.0%	10658.6%	21069.5%	7957.7%	10655.2%	57162.4%
Indian	87.8%	158.3%	196.3%	42.9%	199.9%	657.1%
Malay	1413.6%	5329.3%	4615.2%	2518.2%	5930.7%	19721.5%
Others	1110.7%	73.9%	278.9%	2921.2%	84.2%	829.0%
ER status						
Positive	5957.3%	10859.7%	17056.3%	8259.9%	7639.6%	49554.1%
Negative	4442.7%	6938.1%	12842.4%	5137.2%	9650.0%	38842.4%
Unknown	00%	42.2%	41.3%	42.9%	2010.4%	323.5%
PR status						
Positive	5149.5%	10960.2%	15651.7%	6245.3%	5126.6%	42946.9%
Negative	5250.5%	6737.0%	14146.7%	6950.4%	8745.3%	41645.5%
Unknown	00.0%	52.8%	51.7%	64.4%	5428.1%	707.7%
HER2 status						
Positive	3332.0%	4524.9%	9932.8%	5036.5%	6232.3%	28931.6%
Negative	6967.0%	9753.6%	19263.6%	7856.9%	6935.9%	50555.2%
Equivocal	11.0%	10.6%	00.0%	00.0%	00.0%	20.2%
Unknown	00.0%	3821.0%	113.6%	96.6%	6131.8%	11913.0%
Grade						
1	1211.7%	63.3%	175.6%	1712.4%	52.6%	576.2%
2	3534.0%	5731.5%	7926.2%	3626.3%	4925.5%	25628.0%
3	4846.6%	10960.2%	11237.1%	5036.5%	8041.7%	39943.6%
Unknown	87.8%	95.0%	9431.1%	3424.8%	5830.2%	20322.2%
cT^a^						
T1	54.9%	00.0%	20.7%	53.6%	00.0%	121.3%
T2	3332.0%	2614.4%	3411.3%	3827.7%	00.0%	13114.3%
T3	2221.4%	5832.0%	9832.5%	3424.8%	00.0%	21223.2%
T4	4240.8%	6938.1%	11839.1%	5741.6%	00.0%	28631.3%
Unknown	11.0%	2815.5%	5016.6%	32.2%	192100%	27429.9%
cN^b^						
N0	00.0%	2815.5%	4615.2%	2417.5%	00.0%	9810.7%
N1	00.0%	4323.8%	13143.4%	5540.1%	00.0%	22925.0%
N2	00.0%	2413.3%	4113.6%	2820.4%	00.0%	9310.2%
N3	00.0%	137.2%	4113.6%	2619.0%	00.0%	808.7%
Unknown	103100%	7340.3%	4314.2%	42.9%	192100%	41545.4%
ypT^c^						
Tis	98.7%	52.8%	103.3%	96.6%	00.0%	333.6%
T0	109.7%	95.0%	4615.2%	139.5%	2513.0%	10311.3%
T1	2928.2%	5027.6%	5317.5%	3223.4%	147.3%	17819.5%
T2	3635.0%	7139.2%	11437.7%	3827.7%	2010.4%	27930.5%
T3	1110.7%	3418.8%	6421.2%	3021.9%	168.3%	15516.9%
Unknown	87.8%	126.6%	155.0%	1510.9%	11760.9%	16718.3%
ypN^d^						
N0	5553.4%	6636.5%	13444.4%	3928.5%	6935.9%	36339.7%
N1	2423.3%	5128.2%	7223.8%	3021.9%	5830.2%	23525.7%
N2	1514.6%	3016.6%	6019.9%	3324.1%	2915.1%	16718.3%
N3	98.7%	2714.9%	299.6%	2417.5%	2010.4%	10911.9%
Unknown	00.0%	73.9%	72.3%	118.0%	168.3%	414.5%
Neoadjuvant chemotherapy regimen						
Taxane containing	9188.3%	12267.4%	22173.2%	11986.9%	3920.3%	59264.7%
Nontaxane containing	109.7%	5530.4%	8126.8%	96.6%	15379.7%	30833.7%
Unknown	21.9%	42.2%	00.0%	96.6%	00.0%	151.6%
Surgery type						
Breast‐conserving surgery	2019.4%	3519.3%	134.3%	1410.2%	189.4%	10010.9%
Mastectomy	8380.6%	14580.1%	28694.7%	12389.8%	17490.6%	81188.6%
Unknown	00.0%	10.6%	31.0%	00.0%	00.0%	40.4%
Radiotherapy						
Yes	8077.7%	14278.5%	26487.4%	7856.9%	17289.6%	73680.4%
No	00.0%	2011.0%	309.9%	5640.9%	147.3%	12013.1%
Unknown	2322.3%	1910.5%	82.6%	32.2%	63.1%	596.4%
Adjuvant hormone therapy						
Yes	5755.3%	12267.4%	19965.9%	5943.1%	3719.3%	47451.8%
No	00.0%	4223.2%	9832.5%	7856.9%	9247.9%	31033.9%
Unknown	4644.7%	179.4%	51.7%	00.0%	6332.8%	13114.3%

ER, estrogen receptor; TTSH, Tan Tock Seng Hospital; UMMC, University Malaya Medical Centre

a preneoadjuvant chemotherapy clinical T stage.

b preneoadjuvant clinical N stage.

c postneoadjuvant chemotherapy pathologic T stage.

d postneoadjuvant chemotherapy pathologic N stage.

Vital status was obtained from the hospitals’ medical records and ascertained by linkage to death registries in both countries. Patients were followed up from date of diagnosis until date of death or date of last follow‐up, whichever came first. Date of last follow‐up was 30th June 2014 for KKH, 31st July 2013 for NUH, 16th Jan 2014 for NCCS, 1st January 2014 for TTSH, and 1st March 2013 for UMMC. Based on the above definitions of follow‐up, all the patients in our study have follow‐up information.

### Statistical analysis

Association between clinicopathologic variables and pCR was assessed using the Chi‐square test for univariate analysis and logistic regression for multivariate analysis. Patients were excluded from analysis if pCR (ypT0/is) (*N* = 167) or pCR (ypT0/is ypN0) (*N* = 80) status was not available. Overall survivals of patients with and without pCR were compared using Kaplan–Meier and log‐rank analyses, and further stratified by ER status and tumor grade. Hazard ratio (HR) and corresponding 95% confidence interval (CI) was estimated using Cox proportional hazards model. Only patients with vital status were included in survival analysis (*N* = 829). Two‐tailed *P* < 0.05 was considered as statistically significant. IBM SPSS for Windows version 23.0 (SPSS Inc., Chicago, IL) was used to perform all statistical analysis for this study.

## Results

In total, 4.4% of nonmetastatic patients registered in the hospital‐based registries received neoadjuvant chemotherapy, ranging from 3.1% to 6.6% across different hospitals, and from 1.3% to 10.8% across different stages. Summary of clinical and treatment characteristics of patients who received neoadjuvant chemotherapy from each participating hospital is presented in Table 1. In this study of Southeast Asian women, the median age of the patients was 50. Overall, Chinese made up the majority of the patients (571, 62.4%), followed by Malays (197, 21.5%) (Table 1). Histologically, 495 (54.1%) patients had tumors which were ER positive, 429 (46.9%) were PR positive, and 289 (31.6%) were HER2 positive (Table 1). Only a total of 100 (10.9%) patients eventually underwent breast‐conserving surgeries over the entire study period (Table 1).

Overall, 136 patients (18.1% after excluding patients with unknown pCR) and 121 patients (14.4%) achieved pCR (ypT0/is) and pCR (ypT0/is ypN0), respectively, following neoadjuvant chemotherapy. In univariate analysis, preneoadjuvant chemotherapy clinical T stage, grade of tumor, ER status, and HER2 status were significantly associated with pCR (ypT0/is) status (Table 2). Period of diagnosis, grade of tumor, ER status, HER2 status, and type of neoadjuvant chemotherapy were significantly associated with pCR (ypT0/is ypN0) (Table 2). After adjustment in multivariate analysis, ER and HER2 status were significant predictors for both pCR (ypT0/is) and pCR (ypT0/is ypN0). Patients with grade 3 tumor were significantly more likely to achieve pCR (ypT0/is ypN0) than grade 1 and 2 tumors. Further stratification has shown that pCR rate was highest in patients with HER2‐positive, ER‐negative, and grade 3 tumors (Table 3). For grade 2 and grade 3 tumors of same HER2 status, ER‐negative tumors had higher rate of pCR than ER‐positive tumors. pCR rate increased with higher tumor grade for tumors with similar HER2 and ER status. In subgroup analysis by ER, PR, and HER2 status, patients with ER‐negative, PR‐negative, and HER2‐positive tumors were most likely to obtain pCR than other subtypes (Table 4). A higher pCR rate was noted in patients who received taxane‐containing neoadjuvant regimen after correcting for other factors (Table 2). A sensitivity analysis was performed by removing cases with unknown clinicopathologic data. The results remained similar except for the lack of statistical significance for taxane‐containing regimen and increase in odds ratio for HER2‐positive tumor.

**Table 2:** pCR rates of breast cancer patients who underwent neoadjuvant chemotherapy stratified by patient demographics, clinicopathologic, and treatment information

*** ***	pCR (ypT0/is)(*N* = 748)				pCR (ypT0/is ypN0)(*N* = 835)			
	Yes	No	*P*‐value	Adjusted odds ratio and 95% confidence interval	Yes	No	*P*‐value	Adjusted odds ratio and 95% confidence interval
Total	13618.2%	61281.8%			12114.5%	71485.5%		
Ethnicity			0.983				0.651	
Chinese	8918.5%	39181.5%		Ref	7915.0%	44785.0%		Ref
Malay	2517.1%	12182.9%		1.06 (0.58, 1.93)	2212.7%	15187.3%		1.00 (0.55, 1.82)
Indian	917.6%	4282.4%		1.72 (0.71,4.14)	711.3%	5588.7%		1.09 (0.42, 2.81)
Others	1318.3%	5881.7%		0.96 (0.43, 2.14)	1317.6%	6182.4%		1.10 (0.50, 2.45)
Period of diagnosis			**0.001**				**<0.001**	
1993–2004	1815.7%	9784.3%		Ref	138.7%	13691.3%		Ref
2005–2008	3011.5%	23288.5%		1.19 (0.51, 2.74)	258.3%	27591.7%		1.36 (0.56, 3.27)
2009–2013	8423.8%	26976.2%		1.96 (0.86, 4.43)	7921.5%	28978.5%		**3.43 (1.44, 8.16)**
Unknown	422.2%	1477.8%		1.68 (0.38, 7.37)	422.2%	1477.8%		3.22 (0.71,14.67)
Age			0.557				0.633	
<=34	1022.2%	3577.8%		Ref	1017.2%	4882.8%		Ref
35–44	3120.5%	12079.5%		0.79 (0.30, 2.08)	2917.0%	14283.0%		1.06 (0.42, 2.70)
45–54	5919.6%	24280.4%		0.91 (0.37, 2.27)	5215.3%	28784.7%		1.08 (0.44, 2.62)
55–64	2815.1%	15784.9%		0.58 (0.22, 1.55)	2311.7%	17488.3%		0.70 (0.26, 1.82)
65–74	813.3%	5286.7%		0.44 (0.13, 1.45)	710.9%	5789.1%		0.56 (0.17, 1.84)
>=75	00.0%	5100.0%			00.0%	5100.0%		
Unknown	00.0%	1100.0%		0	00.0%	1100.0%		0
cT^a^			**0.011**				0.316	
T1	216.7%	1083.3%		Ref	216.7%	1083.3%		Ref
T2	2419.5%	9980.5%			2117.1%	10282.9%		
T3	3718.5%	16381.5%		0.66 (0.33, 1.31)	3315.9%	17484.1%		0.71 (0.35, 1.44)
T4	3312.5%	23087.5%		**0.42 (0.21, 0.85)**	2910.7%	24289.3%		**0.45 (0.22, 0.93)**
Tx	4026.7%	11073.3%		1.23 (0.60, 2.51)	3616.2%	18683.8%		1.00 (0.48, 2.08)
Grade			**<0.001**				**<0.001**	
1	11.9%	5198.1%		Ref	00.0%	56100.0%		Ref
2	146.5%	20293.5%			124.9%	23495.1%		
3	4613.7%	28986.3%		1.86 (0.96, 3.61)	4211.5%	32288.5%		**2.14 (1.04, 4.38)**
Unknown	7551.7%	7048.3%		**14.34 (7.19, 28.62)**	6739.6%	10260.4%		**10.95(5.30, 22.59)**
ER status			**<0.001**				**<0.001**	
Positive	419.7%	38190.3%		**0.41 (0.25,0.67)**	326.9%	43393.1%		**0.34 (0.20, 0.56)**
Negative	8627.9%	22272.1%		Ref	8023.2%	26576.8%		Ref
Unknown	950.0%	950.0%		0.65 (0.17, 2.51)	936.0%	1664.0%		0.88 (0.26, 3.02)
HER2 status			**<0.001**				**<0.001**	
Positive	6427.4%	17072.6%		**2.93 (1.77,4.84)**	6023.3%	19876.7%		**2.98 (1.79, 4.98)**
Negative	4911.3%	38388.7%		Ref	418.7%	42991.3%		Ref
Equivocal	00.0%	2100.0%		**3.44 (1.46,8.14)**	00.0%	2100.0%		**3.13 (1.30, 7.54)**
Unknown	2328.8%	5771.3%			2019.0%	8581.0%		
Neoadjuvant chemotherapy regimen			0.150				**0.008**	
Taxane containing	10519.9%	42380.1%		**2.12 (1.16,3.87)**	9517.2%	45882.8%		**2.58 (1.37, 4.87)**
Nontaxane containing	3014.4%	17885.6%		Ref	259.3%	24490.7%		Ref
Unknown	18.3%	1191.7%		0.68 (0.07, 6.99)	17.7%	1292.3%		0.97 (0.10, 9.91)

a preneoadjuvant chemotherapy clinical T stage.

Statistically significant values are formatted in bold.

**Table 3:** pCR rates of breast cancer patients who underwent neoadjuvant chemotherapy stratified by HER2, ER status, and grade

HER2+				
	ER+		ER−	
Grade	pCR (ypT0/is)	pCR (ypT0/is ypN0)	pCR (ypT0/is)	pCR (ypT0/is ypN0)
1	00.0%	00.0%	00.0%	00.0%
2	38.6%	25.1%	417.4%	416.0%
3	815.7%	610.7%	1725.0%	1621.0%
Unknown	853.3%	850.0%	2468.6%	2461.5%

HER2−				
	ER+		ER−	
Grade	pCR (ypT0/is)	pCR (ypT0/is ypN0)	pCR (ypT0/is)	pCR (ypT0/is ypN0)
1	12.4%	00.0%	0–	00.0%
2	21.7%	21.6%	15.9%	15.0%
3	56.1%	44.7%	109.9%	109.3%
Unknown	1127.5%	714.6%	1955.9%	1743.6%

ER, estrogen receptor. –, pCR rate can't be calculate with a zero denominator.

**Table 4:** pCR rates of breast cancer patients who underwent neoadjuvant chemotherapy stratified by ER, PR, and HER2 status

*** ***	pCR (ypT0/is)(*N* = 510)				pCR (ypT0/is ypN0)(*N* = 560)			
*** ***	Yes	No	*P*‐value	Adjusted odds ratio^a^ and 95% confidence interval	Yes	No	*P*‐value	Adjusted odds ratio^a^ and 95% confidence interval
ER+ PR+ and HER2−	125.4%	21094.6%	<0.001	Ref	93.8%	23096.2%	<0.001	Ref
ER+ PR+ and HER2+	811.8%	6088.2%		2.39 (0.82, 7.00)	79.5%	6790.5%		2.74 (0.87,8.69)
ER− PR− and HER2+	3030.3%	6969.7%		**6.35 (2.72, 14.81)**	2925.7%	8474.3%		**7.56 (3.07, 18.65)**
ER− PR− and HER2−	2520.7%	9679.3%		**3.00 (1.28,7.06)**	2317.2%	11182.8%		**3.84 (1.52,9.70)**

ER**,** estrogen receptor.

Statistically significant values are formatted in bold.

a adjusted for ethnicity, age, period of diagnosis, preneoadjuvant chemotherapy clinical T stage, grade, and neoadjuvant chemotherapy regimen.

**Figure 1:**
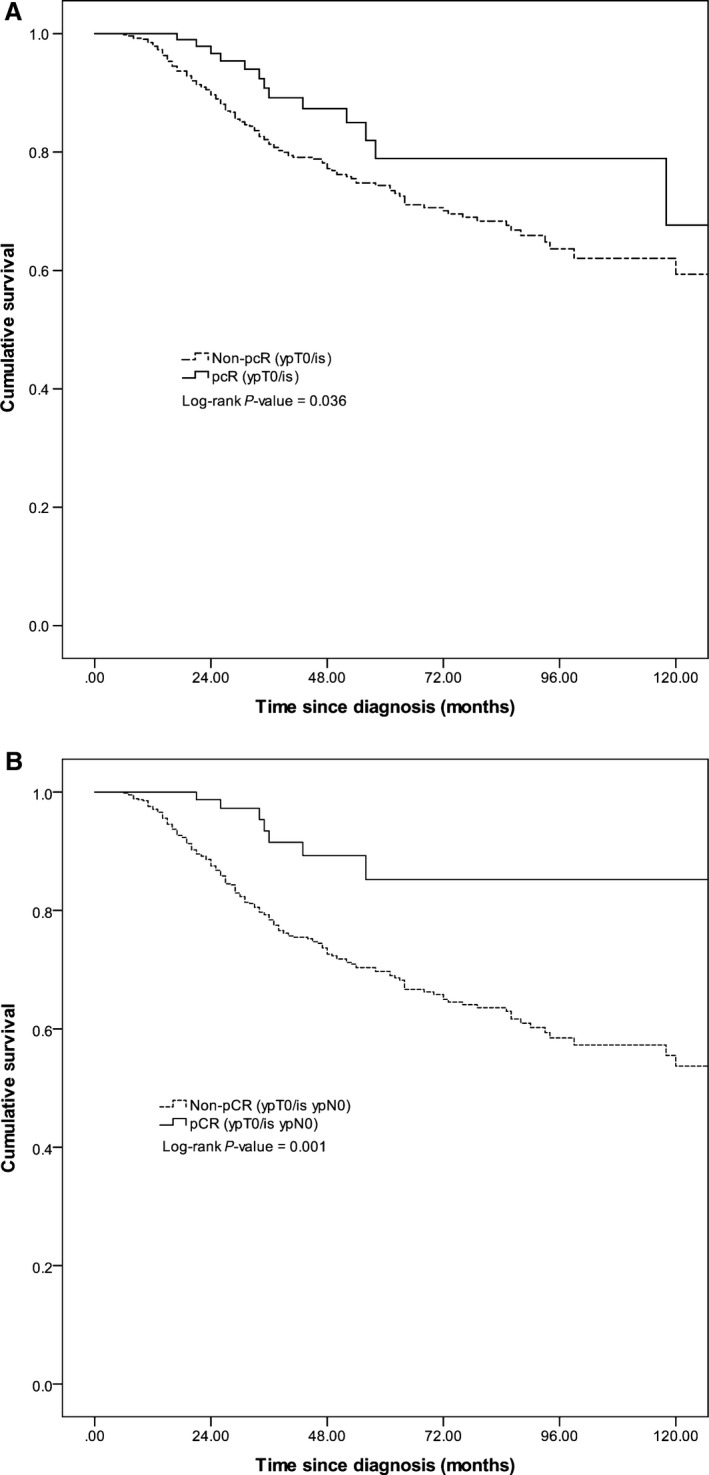
Kaplan–Meier survival curves by (A) pCR (ypT0/is) and (B) pCR (ypT0/is ypN0).

**Figure 2:**
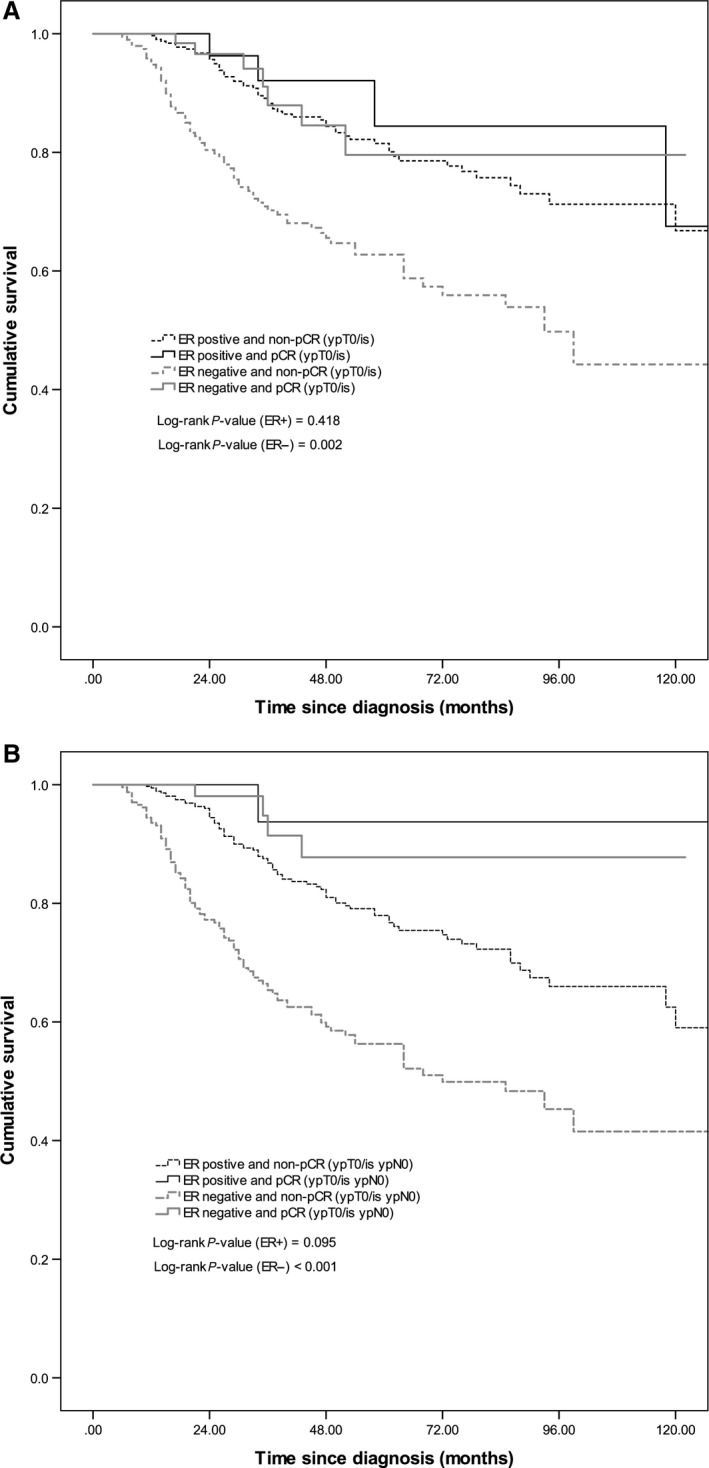
Kaplan–Meier survival curves by (A) estrogen receptor (ER) status and pCR (ypT0/Tis) and (B) ER status and pCR (ypT0/Tis ypN0).

**Figure 3:**
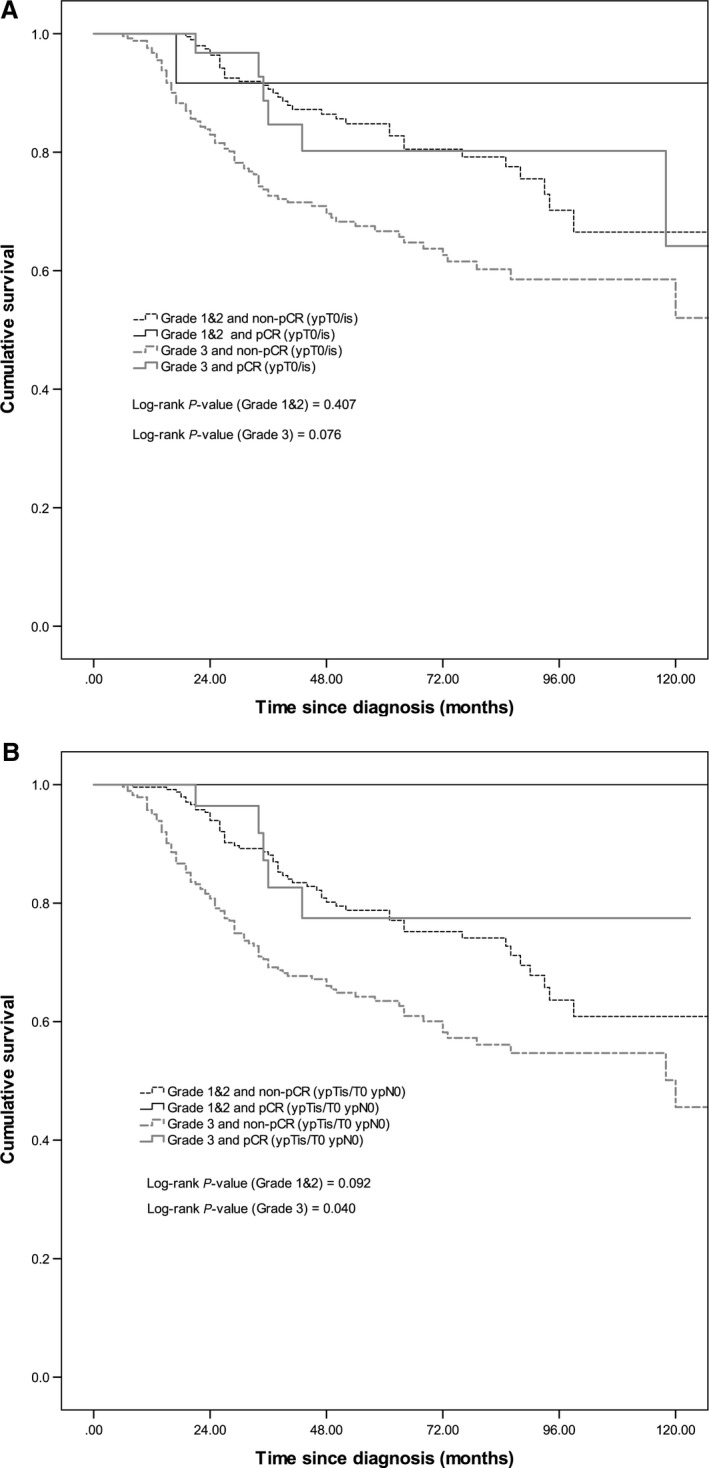
Kaplan–Meier survival curves by (A) tumor grade and pCR (ypT0/Tis) and (B) tumor grade and pCR (ypT0/Tis ypN0).

The median survival of patients receiving neoadjuvant chemotherapy was 11.4 years and overall 5‐year survival was 71.5%. pCR (ypT0/is) (HR = 0.54, 95% CI: 0.31–0.96) and pCR (ypT0/is ypN0) (HR = 0.29, 95% CI: 0.13–0.61) were significant predictors for overall survival (Fig. 1A and 1B). Among patients with ER‐negative tumors, those who achieved pCR (ypT0/is) (HR = 0.30, 95% CI: 0.14–0.66) and pCR (ypT0/is ypN0) (HR = 0.15, 95% CI: 0.06, 0.41) had a significantly better survival (Fig. 2A and B). pCR (ypT0/is) status was not associated with overall survival among patients with ER‐positive tumors (Fig. 2A), grade 1 and 2 tumors, and grade 3 tumors (Fig. 3A). pCR (ypT0/is ypN0) was a significant prognosticator for grade 3 tumors (Fig. 3B) but not for ER‐positive (Fig. 2B) and grade 1 and 2 tumors (Fig. 3B).

## Discussion

In our study population, 4.4% of all nonmetastatic breast cancer patients received neoadjuvant chemotherapy. Although the number of patients diagnosed with breast cancer increased over time, there was no increase in the proportion of nonmetastatic breast cancer patients who were treated with neoadjuvant chemotherapy over the years. pCR rates among breast cancer patients who underwent neoadjuvant chemotherapy were 18.1% (ypT0/is) and 14.4% (ypT0/is ypN0), respectively. Positive HER2 status, negative ER status, and use of taxane‐containing regimen were significant positive predictors for pCR after adjustment for other factors. pCR is associated with better survival among all neoadjuvant patients, and in particular, in patients with ER‐negative tumor.

The incidence of breast cancer is increasing in Asia. As most women present with stage II and above breast cancer 24, neoadjuvant chemotherapy plays an important role in the treatment of breast cancer. Thus far, most pCR rates reported in Asian studies, ranging from 5.9% to 15% 25, 26, 27, were observed from clinical trials of neoadjuvant chemotherapy or single institutional study with very small sample size, which might be different from actual clinical practice. The pCR rate reported in the present study is comparable to results from other observational studies, as well as the National Surgical Adjuvant Breast and Bowel Project (NSABP) B‐18 trial, in which patients received pre‐operative doxorubicin and cyclophosphamide (AC). However, our pCR rate is much lower than those treated with AC followed by docetaxel in the more recent NSABP B‐27 trial 7, 8, 9. The meta‐analysis by Mazouni et al. revealed a similar trend as the NSABP B‐27 trial that patients with both ER‐positive and ER‐negative tumors had higher rate of pCR when taxane are added into the regime 17. As 64.7% of patients received taxane as part of their neoadjuvant regimen in this study, our results may also reflect the difference in clinical profile such as larger inoperable tumor and treatment decision between clinical trials and actual practice.

The distribution of the various races of patients who underwent neoadjuvant therapy in Singapore fits the general distribution of ethnicity of the breast cancer patients in Singapore 28. Chinese patients, as the largest ethnic group in Singapore, were more likely to have breast cancer based on age‐standardized incidence rate and this corresponded to a higher proportion of Chinese who underwent neoadjuvant therapy. However, a closer examination will reveal that the distribution of Malay patients who underwent neoadjuvant therapy for breast cancer is also higher than the distribution of Malay breast cancer patients found in population‐based cancer registry in Singapore (10.9% during 2006–2010) and an earlier published hospital‐based study conducted in Singapore and Malaysia (16% during 1990–2007) 14. Of the patients who had their treatment at UMMC, there was a higher proportion of Chinese as the residents of its catchment were mainly of middle income and Chinese descent, although Malays are the majority ethnic group in Malaysia 14, 29. Previous studies have shown that Malay patients were more likely to present with larger tumor and later stage, as compared to their Chinese counterparts 30. This may result in more Malay patients selected for neoadjuvant therapy.

Patients with worse prognostic tumor profile such as higher grade, ER negativity, and HER2 positivity were found to have better response to neoadjuvant chemotherapy. Specifically, patients with tumor profile of ER negativity, PR negativity, and HER2 positivity had the highest rate of pCR among the four major breast cancer subtypes. This result corresponds to the published findings 21, 31 and is consistent with many other studies and a recent meta‐analysis suggested pCR paradox 32, 33, 34, 35, whereby patients with more aggressive tumors responded better to neoadjuvant chemotherapy. However, given that 54.1% of patients had ER‐positive tumors and 31.6% had HER2‐positive tumors, our pCR rate of 18.1% (ypT0/is) and 14.4% (ypT0/is ypN0) seems to be low. This is likely a result of Asian women having smaller breast size but presenting with higher stage tumors 36. Therefore, neoadjuvant chemotherapy aids in shrinking the size of the tumor instead of directly leading to pCR status.

In our present analysis, pCR is significantly associated with better survival. Subgroup analysis has demonstrated the limitation of pCR for prognostication as pCR is only informative for ER‐negative tumor. This is also observed in other pooled analyses of clinical trials 21, 31.

A meta‐analysis of 14 randomized trials demonstrated that neoadjuvant chemotherapy could reduce mastectomy by 16.6% comparing to adjuvant chemotherapy 37. In this study, even though the rate of pCR is comparable to other countries, the proportion of patients who underwent breast‐conserving surgery after neoadjuvant chemotherapy is noted to be markedly lower (10.9%) than the percentage of 13% to 83% reported in other studies 38. This could be due to smaller breast size among Asian women, larger proportion of advanced‐stage and socio‐cultural factors which may affect patients’ choice between mastectomy and breast‐conserving surgery 39. More studies should be done to find out the reasons for the lower rate of breast‐conserving surgeries in the Asian population.

A strength of the study is its multi‐institutional design which makes our study one of the largest studies done in Asia to determine the demographics of breast cancer patients who underwent neoadjuvant chemotherapy, clinicopathologic predictors for response to treatment, and their long‐term survival in an actual clinical practice setting.

However, the study is not without its limitations. Due to the retrospective nature of the study, some variables were not completely collected for analysis in this study. As regular testing of HER2 was not done before the mid‐2000 in selected hospitals in this study, a proportion of data were missing, and hence, reduced the available sample size for the analysis of the pCR paradox. Grade is more likely to be missing for patients with pCR as no residual tumor was left for pathologic assessment on grade and grade was not commonly evaluated during biopsy in some participating hospitals. This selective loss of data may depend on the value itself as higher grade was more likely to achieve pCR and thus restrict our ability to estimate association between grade and pCR rate. Different cut‐off point for ER status was used for patients from NUH but sensitivity analysis by excluding NUH cases from relevant analyses did not change the interpretation of results.

In conclusion, patients with worse prognostic profile based on ER and HER2 status are more likely to respond to neoadjuvant chemotherapy in the real‐world setting in Asia and pCR is associated with better overall survival especially for patients with ER‐negative tumor.

## Conflict of interest

The authors declare that they have no conflict of interest.
